# Imaging Modalities to Identity Inflammation in an Atherosclerotic Plaque

**DOI:** 10.1155/2015/410967

**Published:** 2015-12-20

**Authors:** Sunny Goel, Avraham Miller, Chirag Agarwal, Elina Zakin, Michael Acholonu, Umesh Gidwani, Abhishek Sharma, Guy Kulbak, Jacob Shani, On Chen

**Affiliations:** ^1^Department of Medicine, Maimonides Medical Center, Brooklyn, NY 11219, USA; ^2^Department of Neurology, Icahn School of Medicine at Mount Sinai, New York, NY 10029, USA; ^3^Department of Cardiology, Icahn School of Medicine at Mount Sinai, New York, NY 10029, USA; ^4^Division of Cardiovascular Medicine, State University of New York Downstate Medical Centre, Brooklyn, NY 11203, USA; ^5^Department of Cardiology, Maimonides Medical Center, Brooklyn, NY 11219, USA

## Abstract

Atherosclerosis is a chronic, progressive, multifocal arterial wall disease caused by local and systemic inflammation responsible for major cardiovascular complications such as myocardial infarction and stroke. With the recent understanding that vulnerable plaque erosion and rupture, with subsequent thrombosis, rather than luminal stenosis, is the underlying cause of acute ischemic events, there has been a shift of focus to understand the mechanisms that make an atherosclerotic plaque unstable or vulnerable to rupture. The presence of inflammation in the atherosclerotic plaque has been considered as one of the initial events which convert a stable plaque into an unstable and vulnerable plaque. This paper systemically reviews the noninvasive and invasive imaging modalities that are currently available to detect this inflammatory process, at least in the intermediate stages, and discusses the ongoing studies that will help us to better understand and identify it at the molecular level.

## 1. Introduction

Atherosclerosis is a progressive inflammatory disease characterized by the accumulation of lipids in the walls of arteries, which over time can result in coronary artery disease, cerebrovascular disease, and peripheral arterial disease [1, 2]. Although there has been an overall increase in the awareness of its risk factors, cardiovascular disease still continues to be the leading cause of death worldwide [3]. Over the last three decades, much work has been done to develop imaging modalities which can diagnose atherosclerosis at least in its intermediate stages and visualize the stage of active inflammation within the vessel wall which converts a stable plaque into an unstable one. It is this process that generates erosion and plaque rupture with subsequent embolization and thrombosis, resulting in acute ischemic events [4]. Inflammation plays a critical role in the formation, progression, and rupture of the atherosclerotic plaque. The hallmark characteristic of inflammation is the presence of macrophages within the plaque lipid core [4, 5]. Ongoing macrophage infiltration and cell death, along with accelerated lipid accumulation, contribute to an enlarging necrotic core that becomes progressively more inflamed, hypoxic, and unstable. Moreover, these cells secrete proinflammatory cytokines (including interleukin-1, monocyte chemotactic protein-1, and tumor necrosis factor-alpha) and matrix metalloproteinase, which actively weaken the fibrous cap, leading to plaque rupture [4]. Detection of atherosclerotic plaques at this inflammatory stage with the use of invasive and noninvasive imaging modalities could allow for the prevention of future cardiovascular events [6].

## 2. Noninvasive Imaging Modalities

Noninvasive imaging techniques do not only visualize the plaque but also could gather data on intraplaque hemorrhage (IPH), plaque inflammation, calcification, and plaque remodelling, thus providing the examiner with information regarding the degree of plaque vulnerability [7]. The noninvasive modalities include computed tomography (CT), magnetic resonance imaging (MRI), ultrasound (US), positron emission tomography (PET), single positron emission computed tomography (SPECT), and microwave radiometry (Table 1).

### 2.1. Computed Tomography (CT)

CT has excellent spatial and temporal resolution, which allows for detailed anatomical delineation of large and medium sized vessels. It is considered as one of the most accurate noninvasive studies for the evaluation of the coronary arteries [8]. With the recent introduction of 16-slice and 64-slice CT scan, enhanced temporal and spatial resolution, and decreased scans times and lower radiation exposure, CT imaging of the coronary tree has truly been revolutionized [9]. A recent study found that the pooled sensitivity and specificity for detecting a greater than 50% stenosis per arterial segment were 93% and 96% for a 64-slice CT, 83% and 96% for a 16-slice CT, and 84% and 93% for a 4-slice CT, respectively [10].

The coronary lesions detected by CT can be divided into calcified, noncalcified, or mixed plaques based on the attenuation of the calcified structures [11]. Detailed imaging of plaque morphology can also be performed when an appropriate contrast medium is used [12]. Contrary to popular belief, the lesions in patients with acute coronary syndrome (ACS) are composed mostly of mixed and noncalcified lesions, which might indicate that the amount of calcification is not an indicator of the vulnerability of the plaque. Studies show that the culprit lesions in acute myocardial infarction (AMI), unstable angina, and stable angina have different calcification patterns. With the help of iodinated contrast agents, even these noncalcified plaques can be detected by CT once the intima results in a 25% narrowing [13].

**Figure 1:**
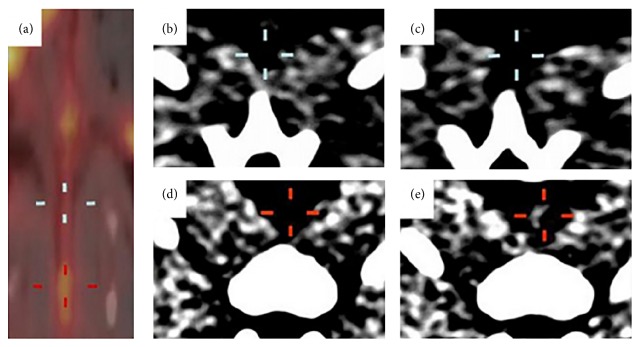
N1177-enhanced CT and corresponding FDG PET from atherosclerotic rabbit. Fused PET/CT coronal view of aorta obtained 3 hours after injection of 18F-labeled fluorodeoxyglucose (FDG) and corresponding axial aortic sections acquired before (b and d) and at 2 hours after injection of N1177, an iodine-based contrast agent that accumulates in macrophages (c and e). Aortic regions with high ((a), red cross) and low ((a), white cross) activities identified with PET at 3 hours after injection of FDG were associated with strong ((e), red cross) and weak ((c), white cross) intensities of enhancement detected in CT at 2 hours after injection of N1177 on corresponding axial views, respectively. Reprinted with permission from Hyafil et al. [20].

Although CT has evolved over the last decade, the hazards of radiation and the use of nephrotoxic contrast agents limit its usage on a large scale [14]. However, with recent technical advances, such as the use of volume scanning (as opposed to helical scanning), one can have a reduction in the effective radiation dose by 90% for the average examination [15]. Recent studies have shown promise with more novel agents, such as iodine-based compounds, gold nanorods with gadolinium, and nuclear tracers, as agents which can both reduce motion artefacts and acquire images with better resolution [16–19]. For example, N1177, a suspension composed of crystalline iodinated particles dispersed with surfactant that has high affinity to activated macrophages, is used, which can detect a vulnerable plaque, and is shown to correlate with FDG uptake (Figure 1) [20].

With the advent of the Coronary Artery Calcium (CAC) score coupled with new methods for decreasing one's exposure to radiation, the future of this imaging modality is promising. Recent studies have used CT imaging as baseline assessment in those about to begin statin therapy [21]. CT imaging is limited in its inability to differentiate stable versus unstable inflamed plaques. However, as this sophistication of this technique continues to improve, its ability to detect and predict atherosclerotic events will continue to advance and may become standard practice in the near future.

### 2.2. Magnetic Resonance Imaging (MRI)

**Figure 2:**
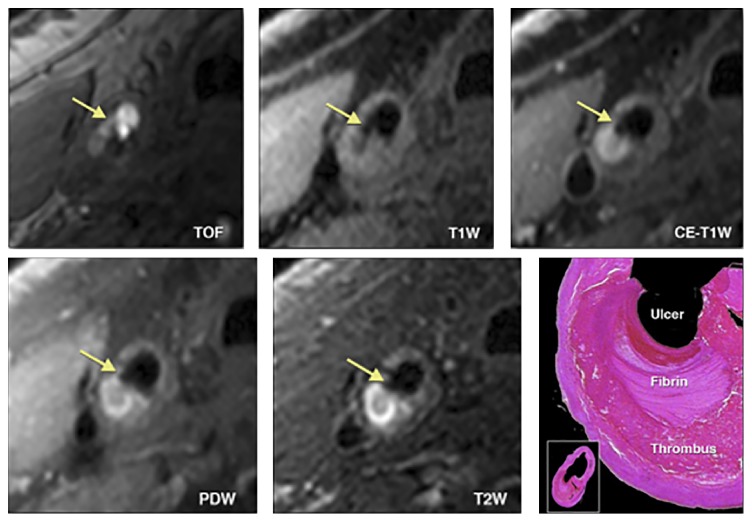
Morphologic characteristics of carotid artery atherosclerosis using MRI. 3-T magnetic resonance imaging (MRI) of a plaque in the right common carotid artery demonstrates fibrous cap rupture with ulcer formation (yellow arrows). The crescent-shaped high-signal region in the proton density-weighted (PDW), T2-weighted (T2W), and contrast enhanced T1-weighted (CE-T1W) images corresponds to a region of thrombus formation, shown on the matched histology section (hematoxylin and eosin stain). Reprinted with permission from Chu et al. [23].

MRI is an accurate and noninvasive imaging modality used for the early detection of atherosclerotic burden in symptomatic patients especially in its intermediate stages when the luminal narrowing is sufficient enough to be detected by the MRI. What separates MRI from other imaging modalities is its ability to visualize plaques undergoing inflammatory changes [22]. It is possible to determine plaque anatomy and composition by using sequences such as T1-weighted, T2-weighted, and proton density-weighted imaging. Multiple imaging sequences may help identify certain plaque morphologies, such as the fibrous cap, the lipid rich necrotic core, intraplaque haemorrhage, neovascularization, and signs of vascular wall inflammation (Figure 2) [23]. Studies have shown that multicontrast magnetic resonance imaging of the human carotid arteries has a sensitivity of 85% and a specificity of 92% when detecting the lipid core and intraplaques haemorrhage [24, 25]. As previously mentioned, an ability to visualize plaques undergoing inflammatory changes would enhance our ability to predict cardiovascular events.

Direct imaging of a thrombus in the coronary artery has been made possible with specially optimized T1-weighted imaging called magnetization-prepared 3-dimensional rapid acquisition gradient echo (MP-RAGE) sequences that are comprised of an inversion recovery radiofrequency (RF) pulse in place of the standard magnetization together with a fast gradient echo acquisition sequence [26]. With the inversion time properly selected, a strong T1-weighting can be achieved, thus effectively detecting haemorrhage inside of an atherosclerotic plaque. An innovative method called the Slab-Selective Phase-Sensitive Inversion-Recovery (SPI) technique is also a promising improvement used in detecting intraplaque haemorrhage [27]. SPI has better intraplaque haemorrhage identification accuracy (*P* < 0.01) and a significantly higher intraplaque haemorrhage-wall contrast-to-noise ratio than MP-RAGE, effectively producing a more enhanced image of what is truly going on inside the plaque [27, 28].

Studies have shown that MRI can detect features of the plaque associated with its vulnerability, including the lipid-rich necrotic core and the thin fibrous cap. An intact fibrous cap is generally seen as a continuous hypointense band against the bright lumen with a smooth surface during T1-weighted imaging, whereas an irregular surface or discontinuity of the hypointense band indicates recent haemorrhage and plaque rupture [29]. Contrast-enhanced MRI (CE-MRI) is typically used to perform magnetic resonance angiography (CE-MRA) and can be supplemented with time-resolved angiography, flow measurement, vessel wall imaging, and plaque characterization for a more comprehensive assessment of vascular diseases [30].

Since the instability in an atherosclerotic lesion is promoted by the activation of mononuclear phagocytes, two MRI strategies have been used to detect this macrophage infiltration. The first technique uses gadolinium to detect the kinetics within the tissue that relate to phagocyte activation and mobilization. The other uses ultrasmall super paramagnetic particles of iron oxide (USPIO) to target macrophages in vivo [31, 32]. The ATHEROMA trial was conducted to test the ability of USPIO to detect plaques in forty asymptomatic patients, demonstrating a significant reduction in plaque uptake with high-dose statin over a 3-month period [33]. Although this trial did not show any significant association between USPIO signal intensity changes and subsequent cardiovascular and cerebrovascular events, it did show that USPIO was an effective method at detecting minute-to-minute changes in the cellular kinetics that were responsible for converting a stable plaque into an unstable one [34]. A recent study has shown that increased vascular permeability using an MR albumin-binding contrast agent and T1-mapping served as a surrogate measure of plaque progression and instability, which has the potential to help stratify atherosclerotic disease progression [35].

The MRI has higher spatial resolution when compared to other imaging modalities such as CT scanning and ultrasound, thus allowing for better tissue contrast. Many novel techniques are being developed at the present time using MRI-specific abilities that will allow us to not only visualize atherosclerotic plaque burden but also actually differentiate inflamed versus noninflamed plaques. This would greatly enhance our ability to determine those individuals who are at greater risk for significant cardiovascular events.

### 2.3. Ultrasound (US)

Doppler ultrasound and high-resolution vascular B-mode ultrasound are widely available and have been shown to accurately depict flow-limiting stenosis in the large arterial circulation [36]. Ultrasound can provide useful information about vulnerable plaque components such as intraplaque haemorrhage, inflammation, lipid core, and vasa vasorum neovascularization, which are all related to plaque vulnerability [37]. However, ultrasound has a relatively poor sensitivity in its ability to detect ulcerations as compared to its ability to detect intravascular irregular borders (sensitivity of 97% and specificity of 81%) [38, 39]. The use of combined echolucency and heterogeneity scales, such as the Gray-Weal scale, may improve diagnostic accuracy but needs further development [40]. The new addition of gas filled microspheres to ultrasound has given the technician the ability to visualize the vasa vasorum and its neovascularization, which are characteristics of vulnerable plaques. The use of microbubble contrast may improve the detection of ulcerations by enhancing the contrast between the lumen and the vessel wall, thus allowing the technician to visualize plaque hemorrhages [41].

Recent evidence suggests that Contrast Enhanced Ultrasonography (CEUS) can be used as a molecular imaging tool to target inflammation and visualize the associated neovascularization in a vulnerable plaque [42]. These two microvascular networks are both involved in the early process of plaque progression and vulnerability and may also be mutually linked with the development of plaque inflammation [42, 43]. CEUS medium consists of microbubbles of gas, enveloped by a shell of different substances (albumin, lipid, polymer, etc.). Gas microbubbles are strong reflectors of acoustic energy, thus increasing the return signal after tissue interrogation with ultrasound. Contrast microbubbles have a diameter of just a few microns (usually <5 micro meter) and have been shown to behave as a true intravascular tracer [44]. CEUS has the ability to image intraplaque neovessels that usually originate from the vasa vasorum in the adventitia which are linked to plaque vulnerability [42]. There have been multiple studies which have confirmed the utility of CEUS in the detection of intraplaque neovascularization with several clinical studies demonstrating that CEUS could help in the differentiation between stable and unstable plaques [42]. Additionally, plaque vascularization measured by CEUS has been shown to correlate positively with 18F-fluorodeoxyglucose (FDG) uptake measured by PET/CT in humans [45]. Although further prospective studies assessing the association of CEUS-detected neovascularization with future cardiovascular events are required, CEUS may represent a promising, safe, and widely available tool for detection of a vulnerable plaque.

### 2.4. Positron Emission Tomography (PET)

**Figure 3:**
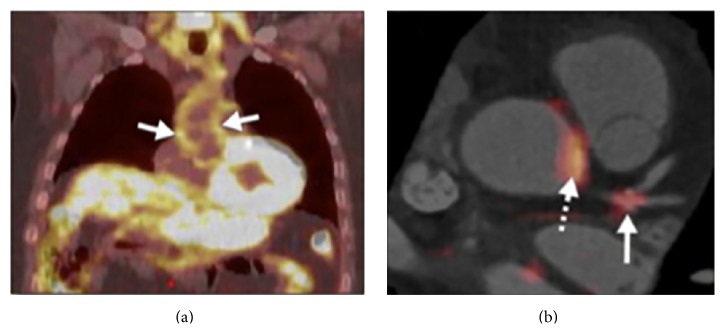
Imaging arterial inflammation using (a) FDG-PET patient demonstrating enhanced aortic uptake of FDG on PET scan, indicating inflammation in the arterial wall due to atherosclerosis. (b) Coregistered FDG-PET/computed tomography images showing FDG uptake at the left main coronary artery trifurcation (solid arrow) in a patient with acute coronary syndrome. Aortic FDG uptake is indicated by the dashed arrow. In such patients, both aortic and coronary artery FDG uptake was increased compared with patients with stable coronary artery disease. Reprinted with permission from Rudd et al. [47].

The underlying principle of nuclear imaging techniques, such as PET, is the use of a radiotracer, which emits gamma rays that can be localized to a cell or receptor in an inflamed plaque. This allows for noninvasive detection of an inflamed and vulnerable plaque prone to rupture in the near future. Following the discovery of fluorine-18-flurodeoxyglucose (FDG), a radiotracer which has increased uptake in carotid plaques in patients with ischemic stroke, several studies have found an association between FDG uptake and vulnerable plaques (Figure 3) [46, 47]. Studies have proven that the amount of FDG uptake is directly proportional to the amount of inflammation in the plaque and that the use of statins reduces the inflammation as determined by the FDG uptake over time [48].

**Figure 4:**
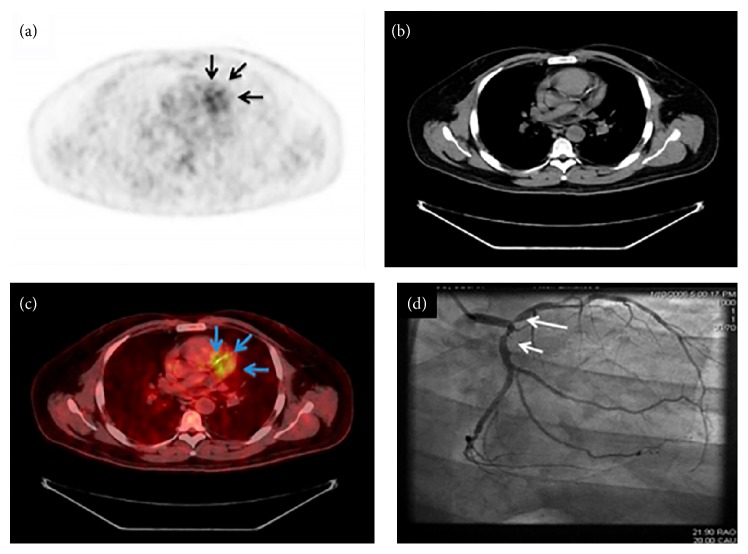
Representative images of coronary tree FDG uptake with corresponding angiographic images. Representative images of the coronary tree FDG uptake (arrows). FDG PET (a). CT (b). PET/CT (c) and coronary angiography (d) from patient with good myocardial uptake suppression with a low carbohydrate, high fat preparation. Reprinted with permission from Wykrzykowska et al. [52].

The disadvantage of FDG-PET is its limited spatial resolution when compared with MRI. Recently, this has been overcome with the use of hybrid imaging techniques such as PET-CT or PET-MRI, which can increase the spatial resolution [49]. Combined positron emission tomography and computed tomography, PET-CT, is a modern noninvasive imaging technique that combines functional information from PET with the fine anatomical detail provided by CT [50]. PET-CT has also been used to assess the efficacy of statin therapy in reducing the level of intravascular inflammation [51]. PET-CT can overcome the challenge of imaging the inflamed atheroma in the coronary vasculature with the use of 18F-FDG due to myocardial uptake of 18F-FDG in the smaller sized coronary arteries. PET-MRI has an advantage over PET-CT in that there is no radiation exposure, and it has a greater ability to differentiate between various plaque components (Figure 4) [52].

Although FDG-PET appears to provide a promising approach for the detection of inflammation in a vulnerable plaque, it does possess certain limitations. First, there are only a limited number of small prospective studies that have shown a correlation between adverse cardiovascular outcomes and increased FDG uptake in atherosclerotic plaque [53]. Second, FDG uptake in a plaque can be influenced by mechanisms other than inflammation such as hypoxia, which may give false positive results [52]. Therefore, even though FDG-PET seems to reliably detect an inflammatory plaque, it cannot currently be used as a predictor of outcome in a given atherosclerotic lesion.

### 2.5. Single Positron Emission Computed Tomography (SPECT)

Both SPECT and PET work on the similar principle of radiofrequency signal uptake, but SPECT uses a different radiotracer which may be Iodine-123, Indium-111, or Technetium-99 [53]. Oxidized LDL labelled to Technetium-99 has been shown to have the greatest sensitivity in detecting vulnerable plaques [54]. Lecithin-like oxidized LDL receptor 1 (LOX-1) is a cell surface receptor for oxidized LDL that has been implicated in plaque instability and ^99*m*^Tc-labeled anti-LOX 1 monoclonal IgG has been shown to have increasing accumulation in a vulnerable plaque, both of which can be detected by SPECT [55]. Another proposed mechanism for the detection of plaque instability with use of SPECT imaging is the detection of apoptotic cells in a plaque with Annexin A5 as the marker for apoptosis. Annexin A5 is a protein which targets the phosphatidylserine surface expression of cells (such as macrophages and platelets) during the apoptotic process. ^99*m*^Tc-labelled Annexin A5 has been shown to have increased uptake in an inflamed atherosclerotic plaque [56, 57].

Despite these promising results, the use of SPECT in the detection of inflammation or plaque instability is limited due to the lack of resolution and low specificity. Additionally, this technique is not cost-effective [58].

### 2.6. Microwave Radiometry (MR)

Microwave radiometry (MR) is a newly developed, noninvasive method, which possesses a high level of accuracy in the detection of the relative changes in temperature of human tissue, thus indicating degree of inflammation within an atherosclerotic plaque [59, 60]. Both experimental and clinical studies have proved the efficacy of microwave radiometry in the detection of vulnerable plaques, with recent studies also demonstrating the association of microwave radiometry with plaque neovascularization as assessed by contrast enhanced ultrasound (CEUS) [61]. After validation of MR in rabbit studies as test subjects, the first application of MR in human carotids was performed to demonstrate its utility in the detection of thermal heterogeneity [62]. Forty-four patients with significant carotid artery stenosis were included in the study. The primary outcomes of this study showed that MR can measure thermal heterogeneity of carotid atheromatic plaques in vivo and that in vivo temperature measurements by MR correlated well with the ultrasound findings of atherosclerotic plaque characteristics [62]. There is no gold standard method for the in vivo quantification of neovascularization and/or inflammation detected by MR; thus more clinical studies are needed.

## 3. Invasive Imaging Modalities

Invasive imaging techniques utilize intravascular catheters that are mounted with an imaging device and have revolutionized our understanding of the atherosclerotic plaque. Invasive imaging modalities are able to provide the highest resolution images with in-depth analysis about vessel wall and plaque morphology [63]. The current intravascular imaging techniques available to assess vulnerable plaques include intravascular ultrasound (IVUS), optical coherence tomography (OCT), intravascular magnetic resonance, and near infrared spectroscopy (NIRS) (Table 2).

### 3.1. Intravascular Ultrasound (IVUS)

**Figure 5:**
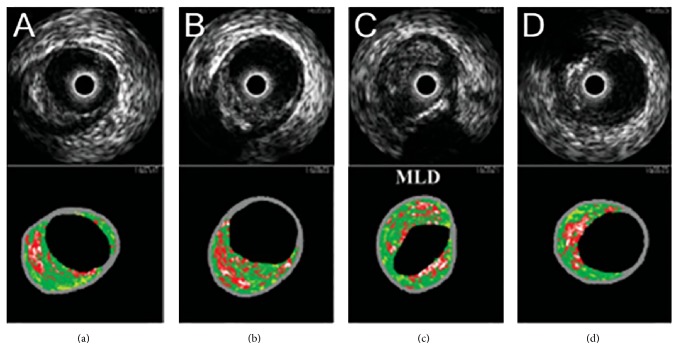
Four cross-sectional images from proximal to distal within the same patient coronary lesion obtained by IVUS and VH. In the upper panels we see grey-scale IVUS with reconstructed IVUS virtual histology in the lower panels. (a) A thick fibrous cap overlying a necrotic core. (b) A thick fibroma can be seen with the thick overlying fibrous cap containing small spots of necrotic core. (c) Minimal Lumen diameter site. (d) A thin-cap fibroatheroma can be seen. Reprinted with permission from Surmely et al. [69].

IVUS consists of an ultrasound unit mounted on the tip of an intravascular catheter, which consists of a piezoelectric material with either a single element or 64 elements. The difference between these units is the frequency range. The single unit frequency is 30–45 MHz and for 64 elements it is 20 MHz. These catheters can provide tomographic images of the vessel, vessel wall, and the atherosclerotic plaque [64]. Since its introduction in 1972, IVUS has advanced from its conventional grey-scale IVUS to its newest version of virtual histology intravascular ultrasound (VH-IVUS), which uses the same equipment and technology as the grey-scale IVUS but with the use of spectral analysis to interpret the back-scattered signals using power spectrum graph which plots the back-scattered US signals against the frequency [65]. Grey-scale IVUS is helpful in determining the vessel lumen size, distribution of the plaque, and severity of the plaque in addition to its ability to detect the plaque cross-sectional area, but it cannot determine the plaque histology [66]. Also, the detection of thin-cap lipid-rich vulnerable plaque (<65 micron) can be difficult to assess via grey-scale IVUS as its resolution is approximately 100 micron [67]. VH-IVUS allows for real time qualification of plaques into different subtypes and effectively overcomes the limitations of the grey-scale IVUS [68]. VH-IVUS uses autoregressive modelling to convert the radiofrequency data into a power spectrum graph. The statistical classification system sorts the radiofrequency data based on the combination of spectral parameters into one of four subtypes: (1) fibrous plaque with a dark green spectral color code, (2) fibrofatty plaque with a yellow-green spectral color code, (3) necrotic core with a red spectral color code, and (4) dense calcium with a white spectral color code (Figure 5) [69].

Pathological studies have shown that the rupture of the thin-cap fibroatheroma (TCFA) is the most common cause of acute thrombotic coronary occlusion and VHIVUS has been able to identify this TCFA with a necrotic core of >10% and without evidence of overlying fibrous tissue [70, 71]. The biggest disadvantage of IVUS is its inability to detect inflammation within a plaque, especially as the inflammatory cells (i.e., macrophages) within the fibrous cap require a resolution of around 10–20 microns for detection, which is not possible using IVUS [72].

### 3.2. Optical Coherence Tomography (OCT)

Optical coherence tomography is an intravascular invasive imaging modality that uses a similar principle as IVUS but instead implements near infrared light instead of ultrasound for imaging [73]. It has ten times higher image resolution and greater tissue contrast as compared to IVUS [74]. OCT is useful in the evaluation of plaque structure due to its resolution, providing the examiner with the ability to analyse various plaque components including fibrous cap thickness, the necrotic core, macrophage infiltration, plaque rupture/erosions, and plaque calcium content. It can also visualize calcified nodules, erosions, and microthrombi near the lumen [75].

**Figure 6:**
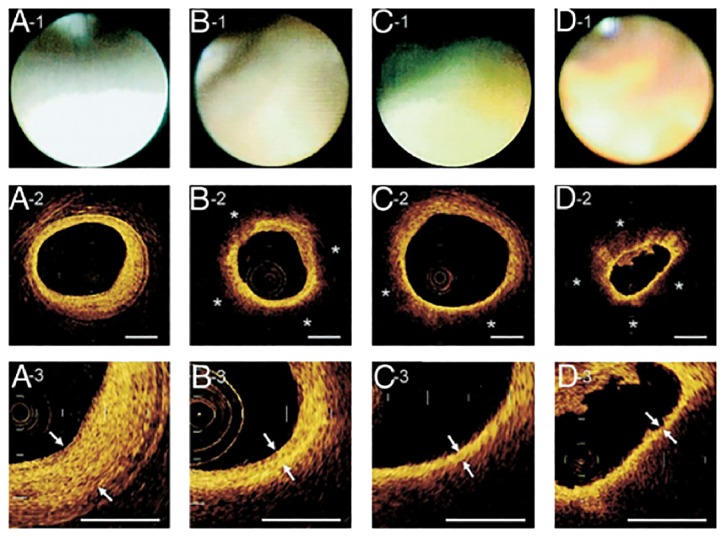
Angioscopic and corresponding OCT images obtained in patients presenting with acute coronary syndrome. In the angioscopic images, plaque color is graded as white (A-1), light yellow (B-1), yellow (C-1), or intensive yellow (D-1). In the optical coherence tomography (OCT) images, a lipid pool (∗) is characterized by a signal-poor region (A-2, B-2, C-2, and D-2). The fibrous cap is identified as a signal-rich region between the coronary artery lumen and inner border of lipid pool in the OCT image, and its thickness is measured at the thinnest part (A-3, B-3, C-3, and D-3; arrows). Reprinted with permission from Kubo et al. [76].

Among all the available intravascular imaging modalities, OCT has been shown to have enough resolution to measure the thickness of the thin fibrous cap, which is the main characteristic of TCFA (Figure 6) [76].

Studies show that OCT imaging provides an accurate measurement of fibrous cap thickness with a mean difference of −24 + 44 micron between the thicknesses measured by OCT versus that measured via digitalized histological images [77]. OCT allows for in-depth analysis of the distribution of inflammatory cells within a vulnerable plaque due to its excellent resolution. Macrophages appear as bright spots with high signal variance from the surrounding tissue because of their lipid content, which contains a high degree of optical contrast [78]. OCT together with ultrasmall super paramagnetic iron oxides [USPIO] can magnify the detection of inflammation in the plaque, thus distinguishing this imaging modality from IVUS [79]. OCT can also detect plaque rupture and erosion, the levels of which differ significantly in different types of unstable angina patients [80]. Additionally, the full extent of OCT imaging can be utilized in conjunction with the use of functionalized magnetofluorescent nanoparticles targeting endothelial markers, such as VCAM-1, which is a critical component of the leukocyte-endothelial adhesion cascade which regulates the atherogenic process [81].

One of the limitations of using short wavelength OCT is the reflection it produces off of the red blood cells, which can diminish the image quality during blood flow and requires frequent saline flushing from the guidewire to clear the image field [82]. This, together with the slow image acquisition speed of current OCT, makes it difficult to detect long arterial segments. However, recent improvements in OCT have rectified this issue, thus allowing for the creation of OFDI (Optical Frequency Domain Imaging) through which images can be obtained at a higher frame rate (>100 frames/sec) with faster 3D image of a long vessel in just one nonocclusive saline flush [83].

### 3.3. Intravascular Magnetic Resonance

The underlying principle in this technique is the utilization of pulsed field gradients together with magnetic resonance imaging, by which the water diffusion coefficient can be calculated. The diffusion coefficient *D* is equal to 0.26 ± 0.13 × 10^−5^ cm^2^·s^−1^ in plaque lipid core, 1.45 ± 0.41 × 10^−5^ cm^2^·s^−1^ in a collagenous cap, and 1.54 ± 0.30 × 10^−5^ cm^2^·s^−1^ in normal media [84]. Water will diffuse less in lipid-rich plaques than in fibrous plaques, which allows us to determine the lipid content in atherosclerotic vessels. This technique can determine the composition of most plaques and could now be performed at the level of the aorta and coronary arteries [85].

Recent data has shown that this new method, when compared to ex vivo histology, showed a sensitivity of 100% and a specificity of 89%. Some of its limitations involve necessity of mechanically rotating the catheter within the vessel. However, much of the recent preliminary data shows that this new method may be superior to the IVUS, though its ability to detect inflammation in an atherosclerotic plaque is yet to be determined [86].

### 3.4. Near Infrared Spectroscopy (NIRS)

This near infrared spectroscopy (NIRS) method is based on the concept of organic molecules absorbing and scattering light differently. As a result, different plaques made up of differing concentrations of lipids and proteins will scatter light differently, thus allowing for a novel technique in which to differentiate atherosclerotic plaque makeup [87]. Recent work with explanted human aorta specimens shows NIRS ability to differentiate between a lipid pool, a thin cap, and inflammatory cells with sensitivity between 77% and 90% and specificity between 89% and 93% [88]. Further studies are needed to establish whether this method will be just as accurate in vivo, as well as its ability to detect inflammation.

## 4. Conclusion and Future Directions

Over the past thirty years, remarkable advances have been made in understanding the role of inflammation in a vulnerable plaque and the sequence of events that make a plaque vulnerable/prone to rupture. It is now widely accepted that inflammation plays a key role in plaque instability, conversion into a vulnerable plaque, and subsequent rupture, thus leading to ischemic events. A number of different novel imaging modalities have been investigated to define the specific characteristics of vulnerable plaque. However, most of these techniques are still undergoing constant refinement and cannot reliably identify vulnerable plaque in the clinical setting. It is important to realize that plaque composition is not equal to plaque vulnerability. Most of the methodologies described in this review are able to detect particular components of plaque, for example, lipids and calcium. However, at present, there is no definitive evidence that in vivo plaque composition is directly related to plaque vulnerability or that the observed characteristics of a plaque are related to outcome. Further research is required to increase the sensitivity and specificity of these modalities to more accurately predict adverse events in the context of high-risk plaque.

Traditionally, scientists and clinicians have only been able to determine the molecular composition of some of the most devastating atherosclerotic plaques by observing and dissecting histological specimens. Currently this practice is changing. In the very near future, the possibility of performing high resolution imaging, both invasively and noninvasively, will allow us to effectively evaluate every component of an inflamed plaque in vivo. As it currently stands, we do possess the ability to invasively pursue certain plaques, but as we move along into the future, the practice will move towards acquisition of noninvasive mechanisms. Using noninvasive techniques and imaging modalities to determine which plaques have become significantly inflamed and unstable will allow the clinician to make real time decisions about what the next step should be to prevent an acute ischemic event.

## Figures and Tables

**Table 1:** Noninvasive imaging modalities to detect a vulnerable plaque.

Noninvasive imaging technique	Spatial resolution	Plaque characteristic identified	Advantages	Limitations
CT	50 micron	Plaque morphology (eccentric pattern, outward remodelling, and spotty calcifications), coronary plaque burden, cap thickness, and macrophages (N1177-specific contrast agent)	High spatial and temporal resolution, real time, quite fast, operator-independent, and excellent calcium detection	Radiation exposure, contrast, difficult to distinguish thrombus, blooming artefacts by calcium, and claustrophobia

MRI	10–100 micron	Plaque morphology, plaque composition, lipid-rich necrotic core, intraplaque haemorrhage, neoangiogenesis, macrophages, flow measurement, and quantification of stenosis	No radiation, high soft tissue contrast, can be repeated over time, functional, operator-independent, with or without contrast, and many plaque components detected	Low resolution, system fibrosis due to contrast agent, time-consuming, metal implants contraindicated, claustrophobia, cardiac motion artefact, and limited spatial resolution

Ultrasound	50 micron	Plaque morphology, intima media thickness, flow velocities, and neoangiogenesis (contrast US)	High temporal resolution, cheap, easy to use, no radiation, bedside/large availability, fastest, and functional	Limited sensitivity and specificity, interobserver variability, calcium and air artefacts, limited spatial resolution, and penetration

PET	1-2 millimeters	Plaque inflammation, macrophages, and neoangiogenesis	High sensitivity and specific targets are detected	Limited resolution, radiation exposure, expensive, limited availability, myocardial uptake of FDG, and cardiac motion artefact

SPECT	0.3–1 millimeters	Plaque inflammation, apoptosis, lipoprotein accumulation, chemotaxis, angiogenesis, proteolysis, and thrombogenicity.	High sensitivity, low cost, and more spatial resolution as compared with PET	Limited resolution, nonspecificity, radiation exposure, limited availability, and cardiac motion artefact

CT, computed tomography; MRI, magnetic resonance imaging; US, ultrasound; PET, positron emission tomography; SPECT, single positron emission computed tomography.

**Table 2:** Invasive imaging modalities to detect a vulnerable plaque.

Invasive imaging techniques	Spatial resolution	Plaque characteristic identified	Advantages	Limitations
IVUS	150–250 micron	Plaque distribution, severity, cross-sectional area, and characterization of plaque (lipid core and spotty calcification)	High resolution images of vessel wall and plaque structure	Intra- and interobserver subjectivity, invasiveness, limited spatial resolution, and limited temporal resolution

OCT	4–20 micron	Plaque composition (fibrous, fibrofatty, and fatty), thin fibrous cap, macrophages, neoangiogenesis, and collagen formation	10 times higher image resolution compared to IVUS and greater tissue contrast	Requires blood-free imaging field, intra- and interobserver variation, invasiveness, and limited tissue penetration

IVMR	120 micron	Early atherosclerosis and more advanced plaque formations and plaque composition (lipid, fibrous, and calcified tissues)	High resolution of plaque structure and composition	Invasiveness and need for occlusion balloon

NIRS	NA	Thin fibrous cap, lipid core, and macrophages	High resolution of plaque structure with reliability	Invasiveness, limited tissue penetration, and cardiac motion artefact

IVUS, intravascular ultrasound; OCT, optical coherence tomography; IVMR, intravascular magnetic resonance; NIRS, near infrared spectroscopy.

## Abbreviations

CT: Computed tomography
MRI: Magnetic resonance imaging
US: Ultrasound
PET: Positron emission tomography
SPECT: Single positron emission computed tomography
MR: Microwave radiometry
IVUS: Intravascular ultrasound
OCT: Optical coherence tomography
NIRS: Near infrared spectroscopy.
